# Whole-genome sequencing reveals high-risk clones of *Pseudomonas aeruginosa* in Guangdong, China

**DOI:** 10.3389/fmicb.2023.1117017

**Published:** 2023-04-14

**Authors:** Yonggang Zhao, Dingqiang Chen, Boyang Ji, Xingju Zhang, Mikkel Anbo, Lars Jelsbak

**Affiliations:** ^1^Department of Biotechnology and Biomedicine, Technical University of Denmark, Kongens Lyngby, Denmark; ^2^Department of Laboratory Medicine, Microbiome Medicine Center, Zhujiang Hospital, Southern Medical University, Guangzhou, Guangdong, China; ^3^Department of Biology and Biological Engineering, Chalmers University of Technology, Gothenburg, Sweden; ^4^BGI-Shenzhen, Shenzhen, China

**Keywords:** *Pseudomonas aeruginosa*, high-risk clone, multidrug resistance, genomic epidemiology, carbapenem resistant

## Abstract

The ever-increasing prevalence of infections produced by multidrug-resistant or extensively drug-resistant *Pseudomonas aeruginosa* is commonly linked to a limited number of aptly-named epidemical ‘high-risk clones’ that are widespread among and within hospitals worldwide. The emergence of new potential high-risk clone strains in hospitals highlights the need to better and further understand the underlying genetic mechanisms for their emergence and success. *P. aeruginosa* related high-risk clones have been sporadically found in China, their genome sequences have rarely been described. Therefore, the large-scale sequencing of multidrug-resistance high-risk clone strains will help us to understand the emergence and transmission of antibiotic resistances in *P. aeruginosa* high-risk clones. In this study, 212 *P. aeruginosa* strains were isolated from 2 tertiary hospitals within 3 years (2018–2020) in Guangdong Province, China. Whole-genome sequencing, multi-locus sequence typing (MLST) and antimicrobial susceptibility testing were applied to analyze the genomic epidemiology of *P. aeruginosa* in this region. We found that up to 130 (61.32%) of the isolates were shown to be multidrug resistant, and 196 (92.45%) isolates were Carbapenem-Resistant *Pseudomonas aeruginosa*. MLST analysis demonstrated high diversity of sequence types, and 18 reported international high-risk clones were identified. Furthermore, we discovered the co-presence of *exoU* and *exoS* genes in 5 collected strains. This study enhances insight into the regional research of molecular epidemiology and antimicrobial resistance of *P. aeruginosa* in China. The high diversity of clone types and regional genome characteristics can serve as a theoretical reference for public health policies and help guide measures for the prevention and control of *P. aeruginosa* resistance.

## 1. Introduction

*Pseudomonas aeruginosa* (*P. aeruginosa*) is an opportunistic pathogen and one of the major causes of nosocomial infections, particularly affecting intensive care units (ICU) and immunocompromised patients, with a very high morbidity and mortality rate (Botelho et al., 2019; Thornton and Parkins, 2023). In addition, *P. aeruginosa* is the most frequent chronic respiratory infection contributing to the pathogenesis of cystic fibrosis (*CF*) and other chronic underlying diseases (Kawalek et al., 2020). The innate and acquired antibiotics resistance mechanism of *P. aeruginosa* includes reduced permeability, antibiotic efflux, expression change, antibiotic modification/degradation, target protection and target modification (including the acquisition of insensitive functional target) (Pang et al., 2019).

Infections caused by multidrug-resistant (MDR) or extensively drug-resistant (XDR) *P. aeruginosa* are extremely difficult to treat (Kawalek et al., 2020). In Asia, the MDR and XDR rates of *P. aeruginosa* nosocomial pneumonia were reported to 42.8 and 4.9%, respectively (Pang et al., 2019). Over the last few decades, the occurrence of epidemic outbreaks caused by antibiotic resistance strains within hospital environments has been given more attention (Talebi Bezmin Abadi et al., 2019). Recent works have provided further evidence that a small group of MDR/XDR global clones are disseminated in many hospitals worldwide (Telling et al., 2018; Hu et al., 2021; Gravningen et al., 2022). The most prevalent of these globally disseminated clones include ST235, ST111, ST233, ST244, ST357, ST308, ST175, ST277, ST654 and ST298, and they have been denominated high-risk clones (Oliver et al., 2015; del Barrio-Tofiño et al., 2020). Furthermore, some types of *P. aeruginosa* strains have developed resistance to carbapenems, called Carbapenem-Resistant *P. aeruginosa* (CRPA). The production of carbapenemases, especially Ambler class B Metallo-β-lactamases (MBLs), is an important mechanism of carbapenem resistance in *P. aeruginosa* (Yoon and Jeong, 2021). Moreover, in the absence of Carbapenemase-mediated resistance, the loss of outer membrane porin *OprD*, together with the overexpression of efflux pumps or *ampC* have been shown to be the main mechanisms leading to carbapenem resistance (Botelho et al., 2019). In China, only a few reports are available on the molecular epidemiology and genome information of *P. aeruginosa* high-risk clones. At the same time, research on Carbapenem-Resistant *P. aeruginosa* (CRPA) together with high-risk clones is even more limited.

Guangdong is the most populous province with the highest domestic migration in China (Xu et al., 2022). This study aims to characterize clinical *P. aeruginosa* strains isolated from two hospitals in Guangdong, in terms of clone diversity, antibiotics resistance profiles, and virulence determinants. Furthermore, the relationship between the carbapenem genes and *P. aeruginosa* high-risk clones will be explored. The outcome of this study will fill the gap related to cultures of *P. aeruginosa* and their associated genome sequences in China. The genetic and phenotypic characteristics of *P. aeruginosa* high-risk clones would provide novel insights into the epidemic and genomic mechanism background of this pathogen in China.

## 2. Materials and methods

### 2.1. Study area

The samples were collected from two-level III (tertiary care) hospitals in Guangdong Province, China, where the patients are heterogeneous. From 2018 to 2020, 212 nonduplicate *P. aeruginosa* isolates were obtained at The First Affiliated Hospital of Guangzhou Medical University and Zhujiang Hospital of Southern Medical University.

### 2.2. Ethics approval

The Ethics Committee of the Zhujiang Hospital of Southern Medical University approved the research on May 30, 2021 (approval no.2021-KY-046-01).

### 2.3. Isolate and clinical data collection

Over 3 years (2018–2020), a total of 212 non-duplicate isolates were selected and distributed over different years, with 55 isolates in 2018, 104 isolates in 2019, and 53 isolates in 2020 (Supplementary Table S1). All isolates were stored in 10% glycerol-containing media and were frozen at −80°C until further use. These isolates originate from different clinical specimens processed by the hospital’s laboratory as part of the microbiological diagnostic routine.

### 2.4. Antibiotic susceptibility testing

Antibiotic susceptibility testing was performed with the Minimum Inhibitory Concentration (MIC) method according to the guidelines of the Clinical and Laboratory Standards Institute (Humphries et al., 2021). The antibiotics tested included piperacillin-tazobactam, ceftazidime, cefepime, imipenem, meropenem, aztreonam, ciprofloxacin, levofloxacin, amikacin, tobramycin and colistin. The minimum inhibitory concentrations (MICs μg/mL) were obtained for each isolate, and the isolates were classified as resistant (R), intermediate (I) and susceptible (S) according to the obtained MIC accordingly. Isolates were designated as multidrug-resistant (MDR) if they were resistant to an antibiotic from ≥3 classes and extensively drug-resistant (XDR) if they were resistant to an antibiotic from ≥6 classes tested following standardized criteria (Magiorakos et al., 2012). This study, Carbapenem-Resistance *P. aeruginosa* (CRPA) was defined as an isolate with imipenem and/or meropenem resistance.

### 2.5. Bacterial identification, DNA extraction and WGS sequencing

Stored strains were regrown from the −80°C stocks using Nutrient agar. The isolates were grown for DNA extraction in Nutrient agar medium overnight at 37°C. DNA was extracted from pure cultures using the MagAttract HMW DNA Kit (Qiagen, Germany) according to the kit protocol. The stLFR technology uses Tn5 transposase for the co-barcoding of DNA libraries. The stLFR library was constructed following the standard protocol using the MGIEasy stLFR Library Prep kit v1.1 (PN: 1000005622) with some process improvement for better assembly of bacterial genomes according to published methods (Wang et al., 2019). The WGS was performed on MGISEQ-2000 platform (DNBSEQ™, MGI, China) according to the manufacturer’s instructions in paired-end mode (PE 100 bp).

### 2.6. Genome assembly and scaffolding

The sequencing reads were evaluated by FastQC 0.11.3, and the reads were filtered by SOAPfilter (v2.2 version) software with the parameters set as follows: -q 33 -y -p -M 2 -f − 1 -Q 10. Athena (v1.3) was used for genome assembly (Bishara et al., 2018). The completeness and pollution of the assembly results were counted by CheckM (v1.0.13), and the SLR-superscaffolder (v0.9.0) was used for the scaffolding. After selection, an average of 5.5 Gb of sequences were kept, with an average of 6.7 million pairs of reads and mean coverage >100X according to the expected genome size (approx. 6.4 Mb). Detailed sequencing statistics and quality results are summarized (Supplementary Table S2). The quality criteria were met for all sequences.

### 2.7. Genotype profiling

The Comprehensive Antibiotic Resistance Database (CARD)^1^ was used as a reference for the antimicrobial resistance genes analysis, and Virulence Factor Database (VFDB)^2^ was used for the virulence factors screening (Alcock et al., 2020; Liu et al., 2022). The serotype was identified by PAst 1.0.^3^ The sequence types of all strains were determined from their whole genome sequence data using the MLST scheme^4^ sited at the University of Oxford (Jolley et al., 2018). The population snapshot of *P. aeruginosa* was inferred by using Phyloviz 2.0. based on the MLST allelic profiles. The minimum spanning tree (MST) analysis was implemented in BioNumerics 8.0 software (Applied-Maths, Sint Maartens-Latem, Belgium). To detect the mutations of the *OprD* gene, the gene sequence encoding the reference protein OprD in *Pseudomonas aeruginosa* strain PAO1 (accession number NC_002516) was obtained and aligned with each target strain by BLASTp script.

## 3. Results

### 3.1. MIC phenotyping

**Figure 1:**
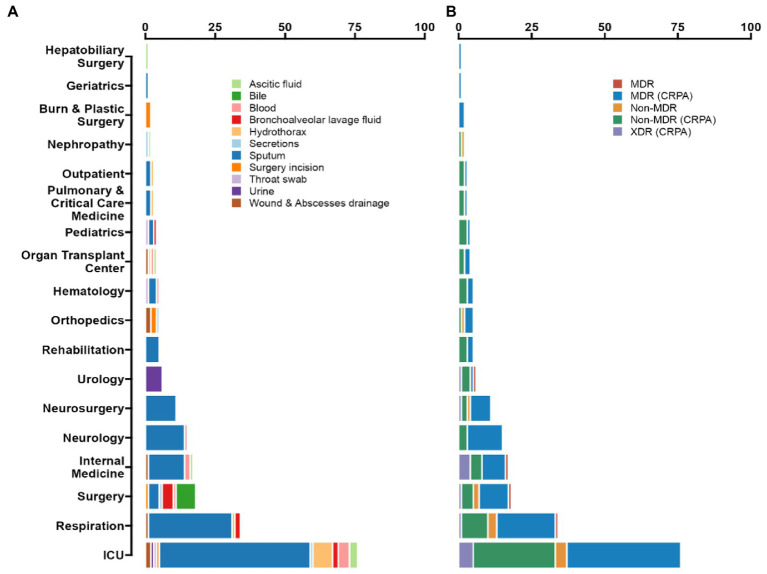
Prevalence of *P. aeruginosa* antimicrobial resistance in Guangdong province, China. **(A)** According to the ward distribution of the studied strains, the number of samples from ICU is 76 (35.85%), Respiratory Department samples numbered 34 (16.04%), and Neurology department samples numbered 15 (7.08%). Among all of the isolated strains, the most frequently isolated specimen was sputum (66.51%), then was hydrothorax (5.19%), blood (4.72%), and bronchoalveolar lavage fluid (BALF) (4.25%). **(B)** Up to 92.45% of the isolates were classified as CRPA, 55.19% as MDR (CRPA) and 6.13% as XDR (CRPA).

The MIC values together with the interpretations are shown (Table 1). 117 strains were MDR (55.19%), and 13 strains were XDR (6.13%) (Figure 1). Among these tested strains, a total of 196 strains belonging to CRPA (92.45%). The identified CRPA strains displayed reduced susceptibilities to most of the tested antimicrobials, but remained highly susceptible to colistin (98.11%) and amikacin (87.74%).

**Table 1:** Resistance rate (percentages) and minimum inhibitory concentrations (MICs) of *P. aeruginosa* isolates in Guangdong (*n* = 212).

Antimicrobial agents	*Pseudomonas aeruginosa* (*n* = 212)				
	S	*I*	*R*	MIC50 (μg/mL)	MIC90 (μg/mL)
Ceftazidime	48.58%	9.91%	41.51%	2	64
Cefepime	53.30%	25.47%	21.23%	8	32
Aztreonam	21.23%	24.06%	54.72%	32	64
Imipenem	11.32%	1.42%	87.26%	16	16
Meropenem	9.91%	13.68%	76.42%	16	16
Piperacillin-tazobactam	29.72%	37.74%	32.55%	32	128
Ciprofloxacin	40.57%	17.45%	41.98%	1	4
levofloxacin	19.81%	9.43%	70.75%	4	8
Amikacin	87.74%	3.30%	8.96%	2	32
Tobramycin	79.72%	0.94%	19.34%	1	16
Colistin	98.11%	0.00%	1.89%	0.5	0.5

MIC_50_ minimal inhibitory concentration inhibiting 50% of isolates, MIC_90_ minimal inhibitory concentration inhibiting 90% of isolates, S susceptible, I intermediately susceptible, R resistant.

### 3.2. Clinical *Pseudomonas aeruginosa* isolates from patients in Guangdong, China

From 2018 to 2020, a total of 212 strains were isolated from patients in Guangdong, China. Among all the isolated strains, the most frequently clinical specimen was from sputum (*n* = 141, 66.51%), then hydrothorax (*n* = 11, 5.19%), blood (*n* = 10, 4.72%) and bronchoalveolar lavage fluid (BALF) (*n* = 9, 4.25%), as shown in Figure 1. According to the ward distribution of the studied strains, the number of samples from the intensive care unit (ICU) was 76 (35.85%), the Respiratory Department samples numbered 34 (16.04%), and the Neurology Department samples numbered 15 (7.08%) (Supplementary Table S1).

Among the 76 strains isolated from ICU, 55 multi-locus sequence types (STs) were identified. The most prevalent STs were ST244 (*n* = 6, 7.89%), ST274 (*n* = 4, 5.26%), and ST381 (*n* = 4, 5.26%). The 34 strains sampled from the ward at the Respiratory Department were classified into 31 STs; only ST253, ST611 and ST703 were with double strains separately. Besides the ICU samples, the intra-hospital transmission, for example, in The First Affiliated Hospital of Guangzhou Medical University, 4 ST569 MDR (CRPA) strains were isolated from the Department of Neurology, and 4 ST1971 strains were purified from the Department of Internal Medicine, which indicates that attention should also be paid to outside-ICUs-acquired-infections.

### 3.3. High sequence type diversity among the studied isolates

Among the isolates studied here, 122 previously reported sequence types and seven novel sequencing types were detected. Most STs are represented only by single isolates (*n* = 93, 43.87%). The high diversity of STs found among the isolates is also evident from the phylogenetic analysis of the strains, as shown in Supplementary Figure S1. There are no clear predominant sequence types among the collected strains, even for the identified multidrug-resistance strains. The most common STs isolated were ST244 (*n* = 9, 4.25%) and ST1971 (*n* = 7, 3.30%), followed by ST381 (*n* = 6, 2.83%) (Supplementary Table S1).

### 3.4. Subclade of the high-risk clones with multidrug resistance

**Figure 2:**
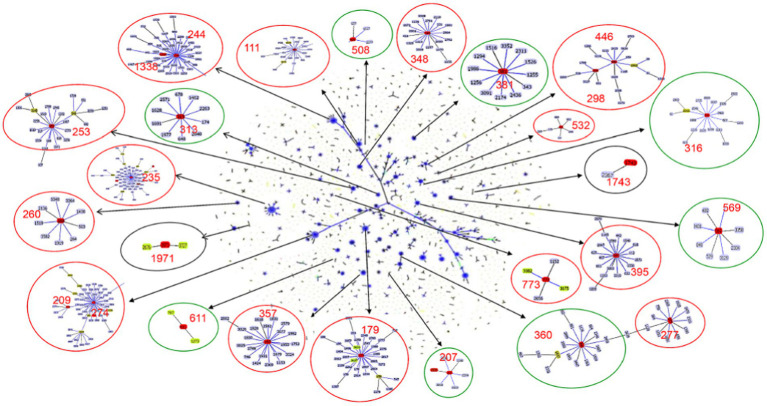
Population snapshot of *P. aeruginosa*. The 3,729 sequence types listed on the *P. aeruginosa* PubMLST database (http://pubmlst.org/paeruginosa, 2022/07/11) are displayed in a single eBURST diagram by setting the group definition to zero of seven shared alleles. Each dot represents a ST, and lines connect single-locus variants. The identified clones in this study were marked in red in each group of related STs. A red circle surrounded the groups reported as international high-risk clones, the groups with the new potential high-risk clones were surrounded by a green circle, and a black circle surrounded the local risk clones identified in this study.

Six global high-risk clones (ST111, ST235, ST244, ST277, ST298 and ST357) (Oliver et al., 2015) were identified in this study (Figure 2). In addition, 12 widely disseminated clones (previously reported as potential high-risk clones) were also identified, including ST108 (Telling et al., 2018), ST179 (Moloney et al., 2020), ST253 (Fischer et al., 2020), ST260 (Martak et al., 2022), ST274 (Sewunet et al., 2022), ST348 (Dix et al., 2022), ST395 (Petitjean et al., 2017), ST446 (Pincus et al., 2019), ST463 (Hu et al., 2021), ST532 (Founou et al., 2020), ST699 (Rada et al., 2021) and ST773 (Singh et al., 2021). We observed that several MDR clones identified in this study were related to high-risk clones and were thus part of the same subclade as them (Table 2). For example, ST446 (*n* = 1) was part of the sub-clade with high-risk clone ST298. Additional examples include ST1338 (*n* = 4, ST244), ST3674 (*n* = 2, ST244), ST209 (*n* = 1, ST274), and ST2326 (*n* = 2, ST207) (Table 2).

**Table 2:** The successful clones identified in this study with potential risks.

Clone types	Description of this study				Reported countries
	Strains No. and percentage	Serotype	MIC	Source	
ST381	6, 2.83%	O2	XDR(CRPA)	Sputum, Surgery incision	Brazil France Ivory Coast Malaysia Poland Russia Spain
ST207	5, 2.35%	O1	MDR/XDR(CRPA)	Sputum, Tissue	Australia Canada France Malaysia Poland Russia Spain
ST313	4, 1.89%	O1	MDR(CRPA)	Hydrothorax, Sputum, Blood	Armenia Australia France Hungary Poland Russia Spain
ST508	4, 1.89%	O3	MDR/XDR(CRPA)	Sputum, Surgery incision, Bile	Australia France The Netherlands
ST569	4, 1.89%	O3	MDR(CRPA)	Sputum	Australia The Netherlands Central African Republic
ST611	4, 1.89%	O5	MDR(CRPA)	Sputum, Blood, Bile	Australia Poland The Netherlands
ST316	2, 0.94%	O11	XDR(CRPA)	Urine, Sputum	Australia France Ivory Coast Senegal
ST360	2, 0.94%	O5	MDR(CRPA)	Sputum	Australia Senegal Ivory Coast Poland

Besides the reported high-risk clones, we found eight groups/subgroups in the population where the founder STs were represented by multiple isolates with MDR/XDR phenotype (Green circles in Figure 2). These including ST381 (*n* = 6, 2.83%), ST207 (*n* = 5, 2.36%), ST313 (*n* = 4, 1.89%), ST508 (*n* = 4, 1.89%), ST569 (*n* = 4, 1.89%), and ST611 (*n* = 4, 1.89%). These successful emerging *P. aeruginosa* clones with MDR/XDR phenotype are summarized (Table 2).

### 3.5. Antibiotics resistance genes profiling

In this study, 19 isolates harbored genes encoding aminoglycoside-modifying enzymes, including aminoglycoside acetyltransferase genes [*aac(6')-IIa*, *aac(6')-Ib4*, *aac(6')-Ib7*, *aac(6')-Ib9*, *aac(6')-Ib10*, and *aac(3)-IId*] and/or aminoglycoside adenylyltransferase genes (*aadA*, *aadA2*, *aadA3*, *aadA7* and *aadA13*). The identified *bla*_OXA_ type genes were composed of *bla*_OXA-50-Like_ genes (*n* = 212, 100%), *bla*_OXA-1-Like_ genes (*n* = 6, 2.83%), *bla*_OXA-10-Like_ genes (*n* = 4, 1.89%) and *bla*_OXA-21-Like_ genes (*n* = 1, 0.47%). In several cases, the strains were carrying multiple β-lactamases. Among them, nine strains (4.25%) simultaneously carried two different *bla*_OXA_ type genes, and one ST1971 strain (0.47%) carried three different *bla*_OXA_ type genes. Furthermore, one *bla*_VIM-2_ gene and two different *bla*_OXA_ genes were found in each isolate of ST235 (*n* = 2) and ST244 (*n* = 1) strains. *bla*_IMP-9/45_, *bla*_KPC-2_, *bla*_CTX-M-13_ and *bla*_CARB-1/3_ co-existed with one/two *bla*_OXA_ type genes were also discovered in different strains (Table 3).

**Table 3:** Horizontally-acquired β-lactamases identified in collected isolates.

β-lactamases			Clone (Strains)
Ambler class	Type	Enzyme	
Class A	CARB	CARB-1	ST1239(1), ST1338(1)
		CARB-3	ST357(1), ST1295(1), ST1338(3), NA(1)
	CTX-M	CTX-M-13	ST16(1), ST1971(2), ST3393(1), ST3660(1)
	KPC	KPC-2	ST463(1)
Class B	IMP	IMP-9	ST292(1)
		IMP-45	ST316(1), ST381(1)
	VIM	VIM-2	**ST235(2), ST244(1)**
Class D	OXA	OXA-1(OXA-1-like)	ST16(1), ST316(1), ST381(1), ST1971(1), ST3393(1), ST3360(1)
		OXA-10(OXA-10-like)	**ST244**(1), ST292(1)
		OXA-21(OXA-2-like)	ST1971(1)
		OXA-50(OXA-50-like)	ST16(3), ST260(2), ST270(2), ST287(1), ST319(1), ST360(2), ST381(6), ST389(1), ST408(1), ST611(4), ST617(1), ST698(2), ST699(1), ST708(1), ST903(1), ST1123(1), ST1129(1), ST1667(1), ST1718(2), ST1930(1), ST1966(1), ST2211(1), ST2447(1), ST2920(1), ST3393(1), ST3658(1), ST3660(1), ST3661(1), ST3664(1), ST3665(1), ST3667(1), ST3672(1), ST3679(1), ST3682(1), ST3684(2), ST3686(1), ST3690(1), ST3692(1), ST3693(2), ST3693(1), ST3694(1), ST3695(2), NA(3)
		OXA-246(OXA-10-like)	**ST235(2)**
		OXA-486(OXA-50-like)	ST252(1), ST266(2), ST274(5), ST412(1), ST463(1), ST508(4), ST553(2), ST567(1), ST644(1), ST703(2), ST807(1), ST1295(1), ST1655(1), 1748 (1), ST3680(1), NA(1)
		OXA-488(OXA-50-like)	ST235(2), ST253(5), ST313(3), ST377(1), ST560(1), ST606(1), ST3713(1), NA(1)
		OXA-846(OXA-50-like)	**ST111(2),** ST168(1), ST207(5),ST241(2), ST273(1), ST**298(1), ST**313(1), ST316(2), ST**357(5),** ST395(1), ST446(1), ST645(1), ST773(4), ST927(2), ST1021(1), ST1086(1), ST1129(1), ST1203(1), ST 1239 (1), ST1248(1), ST1743(4), ST1920(1), ST1971(7), ST2326(2), ST3659(1), ST3675(1), ST3683(1), NA(1)
		OXA-850(OXA-50-like)	ST27(1), ST108(1), ST155(1), ST179(1), ST209(1), ST**244(9),** ST245(1), ST261(1), ST**277(4),** ST292(1), ST348(1), ST385(1), ST455(1), ST554(1), ST569(4), ST769(1), ST798(1), ST980(1), ST1247(1), ST1338(3), ST1652(1), ST1684(1), ST2069(1), ST2957(1), ST3213(1), ST3666(1), ST3670(1), ST3674(2), ST3677(1), ST3685(1), ST3687(1), ST3688(1), ST3691(1)
		OXA-906(OXA-50-like)	ST532(3)

The bold values represent observed global *P. aeruginosa* high-risk clones.

As with high prevalence rates of *bla*_OXA_ type genes, 211 isolates harbored *Pseudomonas*-derived cephalosporinase (PDC) β-lactamase genes, which includes 24 *bla*_PDC_ types. The dominant type was *bla*_PDC-3_ (*n* = 51), followed by *bla*_PDC-5_ (*n* = 42), which both belong to the extended-spectrum β-lactamase (ESBLs). We found *bla*_PDC_ types and STs had special correspondence relationships. Among them, 7 ST244 strains were carrying *bla*_PDC-1_ and *bla*_PDC-5_, 5 ST357 strains were with *bla*_PDC-11_, and 4 ST277 strains were with *bla*_PDC-5_. Besides high-risk clones, 5 ST253 strains were with *bla*_PDC-68_, 4 ST313 strains were with *bla*_PDC-105_, 6 ST381 strains were with *bla*_PDC-14_, 5 ST207 strains were with *bla*_PDC-337_, and 7 ST1971 strains were with *bla*_PDC-3_, and so on (Supplementary Table S3).

**Figure 3:**
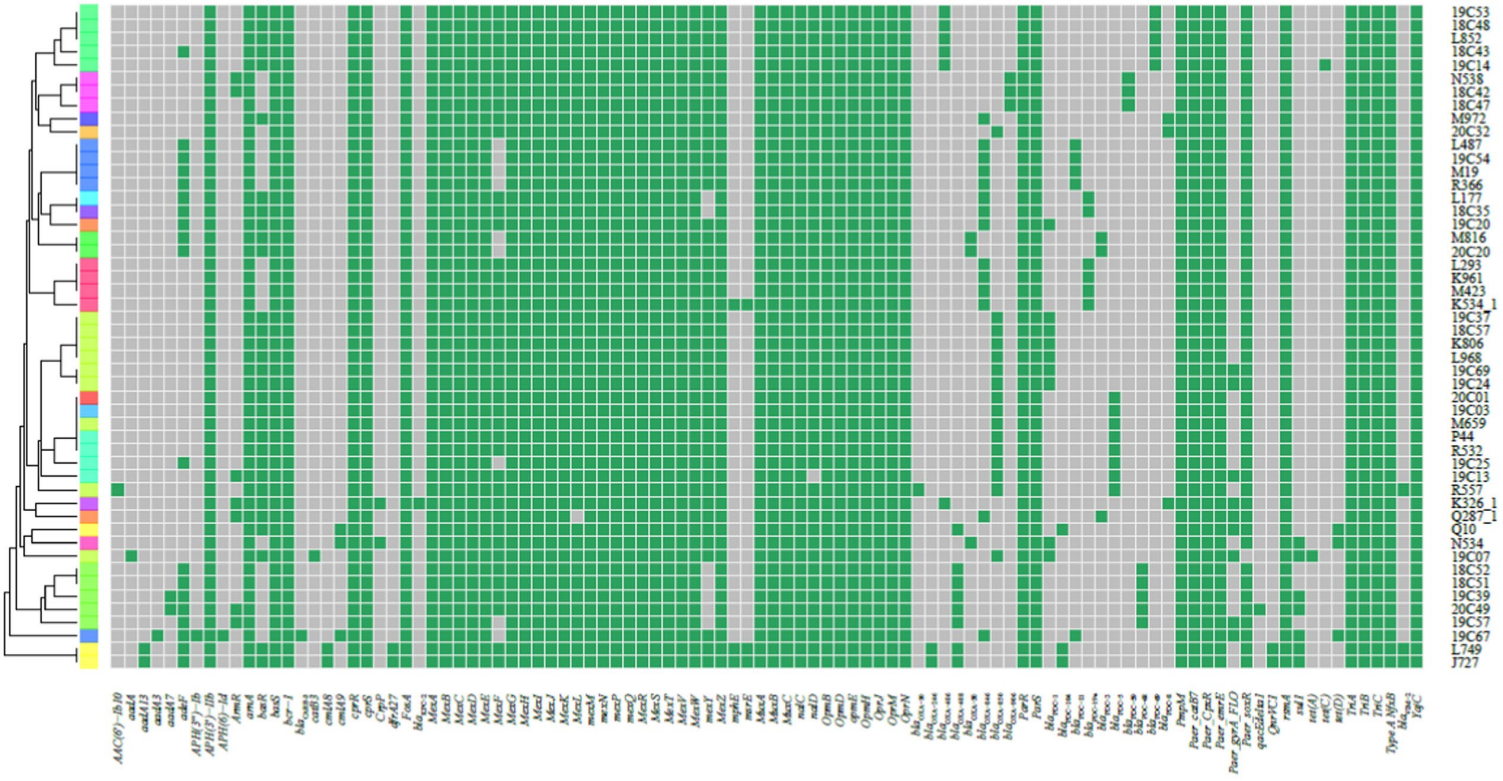
Heat map showing the resistance genes for the collected *P. aeruginosa* high-risk clone strains. The heat map was formed by performing tblastn searches using the sequences present on the Comprehensive Antibiotic Resistance Database (CARD) as queries and the genomes as subjects. Grey indicates the absence, and green indicates the presence of the resistance genes.

We did not find Class A and Class B antibiotic resistance genes (ARGs) in high-risk clone ST111, ST277 and ST298, but the *Klebsiella pneumoniae* carbapenemase (KPC) gene *bla*_KPC-2_ was found in one ST463 strain. The Verona Integron-encoded Metallo-β-lactamase (VIM) gene was detected in one ST244 strain, and two ST235 strains carried two *bla*_VIM-2_ genes in each of the isolates (Table 3). Furthermore, two types of *qnrVC* variants were detected in 6 CRPA strains (2.83%) with five different STs, which included five strains carrying *qnrVC1* and one strain carrying *qnrVC6*. The heat map showing unique resistance genes for identified *P. aeruginosa* high risk clone strains is illustrated (Figure 3). In order to further explore the reasons for the high proportion of CRPA in the collected strains, the *OprD* gene alignment was also performed. BLASTP analysis showed that the wild-type *OprD* gene was not detected in all collected strains (*oprD* mutation strains, *n* = 212).

The *exoU* and *exoS* genes, which encoding effector proteins of type III secretion system (T3SS) in *P. aeruginosa,* were investigated. The more frequent distribution of *exoS* (*n* = 150, 70.75%) was observed compared with *exoU* (*n* = 57, 26.89%). More interestingly, we found the co-presence of both genes in 5 strains (ST463, *n* = 1; ST273, *n* = 1; ST3658, *n* = 1; ST274 *n* = 2).

### 3.6. The antibiotics genes profiling and phenotype correlation

Several class A and class B β-lactamases were identified in 22 strains (10.37%), and all these were classified as CRPA and with multidrug resistance. 8 *bla*_CARB-3_ genes carried strains were identified, and 4 of these strains belonged to ST1338 with XDR phenotype. 5 strains harboring the *bla*_CTX-M-13_ gene were identified and represented multidrug resistance. Isolates producing *bla*_KPC_ type enzymes often relate to the resistance to most of β-lactams and non-β-lactams antibiotics. 1 ST463 strain was carrying *bla*_KPC-2_ gene and represented XDR (CRPA) phenotype. 1 *bla*_IMP-9_ and 2 *bla*_IMP-45_ carried isolates were distributed into three dissimilar STs, including ST292, ST316 and ST381, which were isolated from the same hospital but in different wards. The MIC profiling showed that these three strains were all representing XDR (CRPA) phenotype. Three *bla*_VIM-2_ harboring high-risk clone strains were identified which were distributed in 2 ST235 and 1 ST244 strains. The responding phenotypes were multidrug resistance. Contrary to the high proportion of CRPA, class A and B carbapenemase genes identified in collected isolates are less frequent, which means their reduced susceptibility to carbapenems resulted from various non-enzymatic mechanisms (Figure 3).

## 4. Discussion

The emergence and increase of high-risk isolates, including their antibiotic resistance, has been recognized as a threat for human health (Oliver et al., 2015; del Barrio-Tofiño et al., 2020). However, our understanding of these clones’ global distribution and evolution is still incomplete due to uneven sampling in different regions of the world. Here, we investigated clinical *P. aeruginosa* clones isolated in Guangdong, China. The MLST analysis showed high genetic diversity among collected isolates in agreement with prior studies in China (Fan et al., 2016). Importantly, some international high-risk clones as well as new widely distributed clones (regarded as potential risk clones), were identified in our study. The observations of potential high-risk and locally distributed clones underscore the relevance of continued inspection of the population structure in clinical settings in different parts of the world.

Although previous studies had showed that high-risk clones were predominant among multidrug-resistance strains (del Barrio-Tofiño et al., 2020; Hu et al., 2021; Teo et al., 2021; Aguilar-Rodea et al., 2022), we found that 50/212 isolates (23.58%) belong to the 18 previously reported high-risk clones, and that the remaining isolates showed high diversity together with other reported STs in all strains.

Among the high-risk clones found in our study, we observed that ST111 included two MDR/non-MDR strains from different hospitals. ST235 is the most widely spread *P*. *aeruginosa* high-risk clone associated MDR/XDR isolates and carries various resistance genes (Treepong et al., 2018; Tahmasebi et al., 2022). Three ST235 MDR strains were isolated from the neurology ward, of which two were carrying the *bla*_VIM-2_ gene. The most prevalent high-risk clone in all isolated strains was ST244 (*n* = 9); six multidrug resistance strains were collected from the ICU, of which one harbored the *bla*_VIM-2_ gene. ST277 was reported to be highly prevalent in Brazil, and commonly associated with MBL SPM-1 (Galetti et al., 2019; Silveira et al., 2020). We found ST277 isolates composed of 4 MDR/XDR strains, but the specific MBLs genes were not identified. ST357 with *bla*_IMP_ and *bla*_VIM_ type MBLs was previously reported in Turkey (Çekin et al., 2021) and Czech (Papagiannitsis et al., 2017), three ST357 stains identified in our study showed MDR/XDR phenotype, and one of them harbored *bla*_CARB-3_ gene. ST298 is the subclade of the CC446 clonal complex (Pincus et al., 2019). One ST298 isolate and one ST446 isolate, both with MDR/XDR phenotype, were identified in different hospitals. In some regional research projects, the clonal diversity of *P. aeruginosa* is much lower among multidrug-resistant isolates (del Barrio-Tofiño et al., 2020; Hu et al., 2021; Teo et al., 2021; Aguilar-Rodea et al., 2022). This is not entirely the case among our MDR subsets from 2018–2020. We have observed 84 (39.62%) different STs in the 130 MDR/XDR strains. However, only 12.26% (*n* = 26) of MDR/XDR isolates are actually related to the reported international high-risk clones.

We note that our study is focused on the Guangdong Province. This region is the most populous province with the largest population inflow in China. We speculate that trade and mobility in this region have contributed to the high level of diversity observed among the isolated strains. Overall, our survey of regional *P. aeruginosa* high-risk clones in China may assist in a better understanding of the evolution of these important clone types.

Besides the high-risk clones, eight founder STs of clonal groups/subgroups have been identified (Figure 2), and these founder STs have been reported to be distributed in at least three different countries (Table 2). Furthermore, one special disseminated and multidrug-resistant clone, ST1971 (*n* = 7, 3.30%), has only been identified in China (Wang et al., 2017). This clone can be defined as a regional epidemic risk type for nosocomial healthcare. Some of the STs identified in this study have been previously described as new potential high-risk clones, including ST108 (Telling et al., 2018), ST179 (Moloney et al., 2020), ST253 (Fischer et al., 2020), ST260 (Martak et al., 2022), ST274 (Sewunet et al., 2022), ST348 (Dix et al., 2022), ST395 (Petitjean et al., 2017), ST446 (Pincus et al., 2019), ST463 (Hu et al., 2021), ST532 (Founou et al., 2020), ST699 (Rada et al., 2021) and ST773 (Singh et al., 2021).

The antibiotic profiles of all isolated *P. aeruginosa* strains were quantified by MIC assays. 196 strains (92.45%) were found to be CRPA, and most of them represented resistance to multiple antibiotics (126/196, 64.29%). It was previously reported that meropenem and imipenem resistance rates for *P. aeruginosa* range from 21.6 to 25.6% in China (Wang and Wang, 2020), and the detection rate of CRPA in Guangdong was 41.9, 17.9 and 19.2% from 2018 to 2020, respectively. The CRPA rates determined in this study were higher than the previous studies. Previous studies reported that the CRPA isolating rate of inpatients and those admitted to an ICU is higher than outpatients and those in non-ICU wards (Gill, Nicolau, and on behalf of the ERACE-PA Global Study Group, 2022). Here, 35.85% of strains were isolated from ICU patients, which might explain the high prevalence of CRPA in the collection. In future studies, collecting strains from more diverse wards and improving the randomness of the evaluation would be beneficial.

In contrast to the high rate of carbapenem resistance, the MBLs genes carrying strains were with very low proportion (*n* = 6, 2.83%) in all collected isolates. Only 3 *bla*_IMP_ harbored strains and 3 *bla*_VIM-2_ harbored strains were found. The *oprD* gene has been reported as the main determinant of imipenem resistance (Quinn et al., 2022). The MIC data show that the imipenem resistance rate is 87.26%. The wild-type *oprD* gene was not found in any newly sequenced strains, which implied that the mutational inactivation of the *oprD* gene may play a role in carbapenem resistance. However, the relationship between the mutations in the *oprD* gene and its function cannot be fully understood only by whole genome sequencing, and future investigations should focus on mechanistic studies of these mutations.

Some variants of PDC enzymes (class C β-lactamases) are responsible for the resistance to imipenem and newer β-lactam/β-lactamase inhibitor combinations such as ceftolozane-tazobactam (Papp-Wallace et al., 2020). Furthermore, the production of class D β-lactamases (oxacillinase; OXA) can lead to third-generation cephalosporins or multiple-drug resistance (Vrancianu et al., 2020). In this study, PDC β-lactamase and OXA β-lactamase genes were prevalent in collected strains, which can induce multidrug resistance to some extent by antibiotic inactivation. Previous studies showed that the presence of *qnrVC* genes plays important roles in quinolone resistance (Liu et al., 2018; Saki et al., 2022), and 2.3% of collected *P. aeruginosa* isolates from Guangdong carried *qnrVC* genes (Lin et al., 2020), which is consistent with this study.

The type III secretion system is an important virulence factor of *P. aeruginosa*, contributing to microbial cytotoxicity and invasion (Horna and Ruiz, 2021). The *exoU* and *exoS* genes encode type III secretion virulence effector proteins. Previous studies have shown the mutual exclusion of the *exoU* and *exoS* genes (Bradbury et al., 2010; Juan et al., 2017). Through the VFDB analysis, we found the co-presence of both genes in 5 clone types strains (ST273, ST274, ST463, and ST3658). The *exoS*^+^/*exoU*^+^ strains have no significant differences in the resistance phenotype, but the strains carrying two virulence genes were all classified into XDR (CRPA).

## 5. Conclusion

*Pseudomonas aeruginos*a isolates from hospitals in this study exhibited multidrug resistance to various drugs and a high rate of resistance against carbapenem. MLST analysis further revealed the genetic diversity of these clinical isolates of *P. aeruginosa*. The stLFR technology was first applied for the bacteria antibiotic gene profiling, and the findings indicate that the sequence clone types are independent of carbapenem resistance or MDR/XDR phenotype. The strains harboring *bla*_OXA_ and *bla*_PDC_ genes mainly circulate in the hospital environments studied. The spectrum of high-risk clones is also different from previous reports and has obvious regional characteristics. This means that the control measures for the circulation and success of high-risk clones and CRPA must have a unified global standard and custom local strategies.

## Data availability statement

The datasets presented in this study can be found in online repositories. The names of the repository/repositories and accession number (s) can be found in the article/Supplementary material.

## Ethics statement

The study was approved by the Ethics Committee (full name: The Ethics Committee of the Zhujiang Hospital of Southern Medical University), the reference number 2021-KY-046-01. The samples were obtained with written informed consent and reviewed by the ethical board in accordance with the tenets of the Declaration of Helsinki.

## Author contributions

YZ designed the experimental plan, analyzed WGS data, and finished the draft manuscript writing. DC collected strains and participated in project design. BJ prepared MLST distribution and digital chart. XZ contributed to manuscript revision. MA participated in project design. LJ contributed to conceptualization, project administration, and manuscript revision. All authors participated in the manuscript critical review and editing, and agreed with the above contribution details.

## Funding

This work was supported by a research grant to LJ from the Independent Research Fund Denmark (9039-00350A).

## Conflict of interest

The authors declare that the research was conducted in the absence of any commercial or financial relationships that could be construed as a potential conflict of interest.

## Publisher’s note

All claims expressed in this article are solely those of the authors and do not necessarily represent those of their affiliated organizations, or those of the publisher, the editors and the reviewers. Any product that may be evaluated in this article, or claim that may be made by its manufacturer, is not guaranteed or endorsed by the publisher.

## Footnotes

^1^ https://card.mcmaster.ca/

^2^ http://www.mgc.ac.cn/VFs/

^3^ https://cge.cbs.dtu.dk/services/PAst/

^4^https://pubmlst.org/paeruginosa/; last accessed July 11, 2022.
