# Supplementary material for: Whole-genome sequencing reveals high-risk clones of Pseudomonas aeruginosa in Guangdong, China
Source: Front Microbiol. 2023 Apr 14;14:1117017. doi: 10.3389/fmicb.2023.1117017 (PMC10140354; doi:10.3389/fmicb.2023.1117017)
Supplement: Supplementary file 1 [file Data_Sheet_1.docx]

Supplementary Material

Whole-Genome Sequencing Reveals High-risk Clones of *Pseudomonas aeruginosa* in Guangdong, China

**Yonggang Zhao^1†^, Dingqiang Chen^2†^, Boyang Ji^3^, Xingju Zhang^4^, Mikkel Anbo^1^, Lars Jelsbak^1*^**

^1^Department of Biotechnology and Biomedicine, Technical University of Denmark, Denmark.

^2^Microbiome Medicine Center, Department of Laboratory Medicine, Zhujiang Hospital, Southern Medical University, Guangzhou, Guangdong, 510282, China.

^3^Department of Biology and Biological Engineering, Chalmers University of Technology Göteborg, Sweden.

^4^BGI-Shenzhen, Shenzhen 518083, China.

**^†^**Yonggang Zhao and Dingqiang Chen were equal contribution and first authorship.

*** Correspondence:**

Lars Jelsbak: lj@bio.dtu.dk


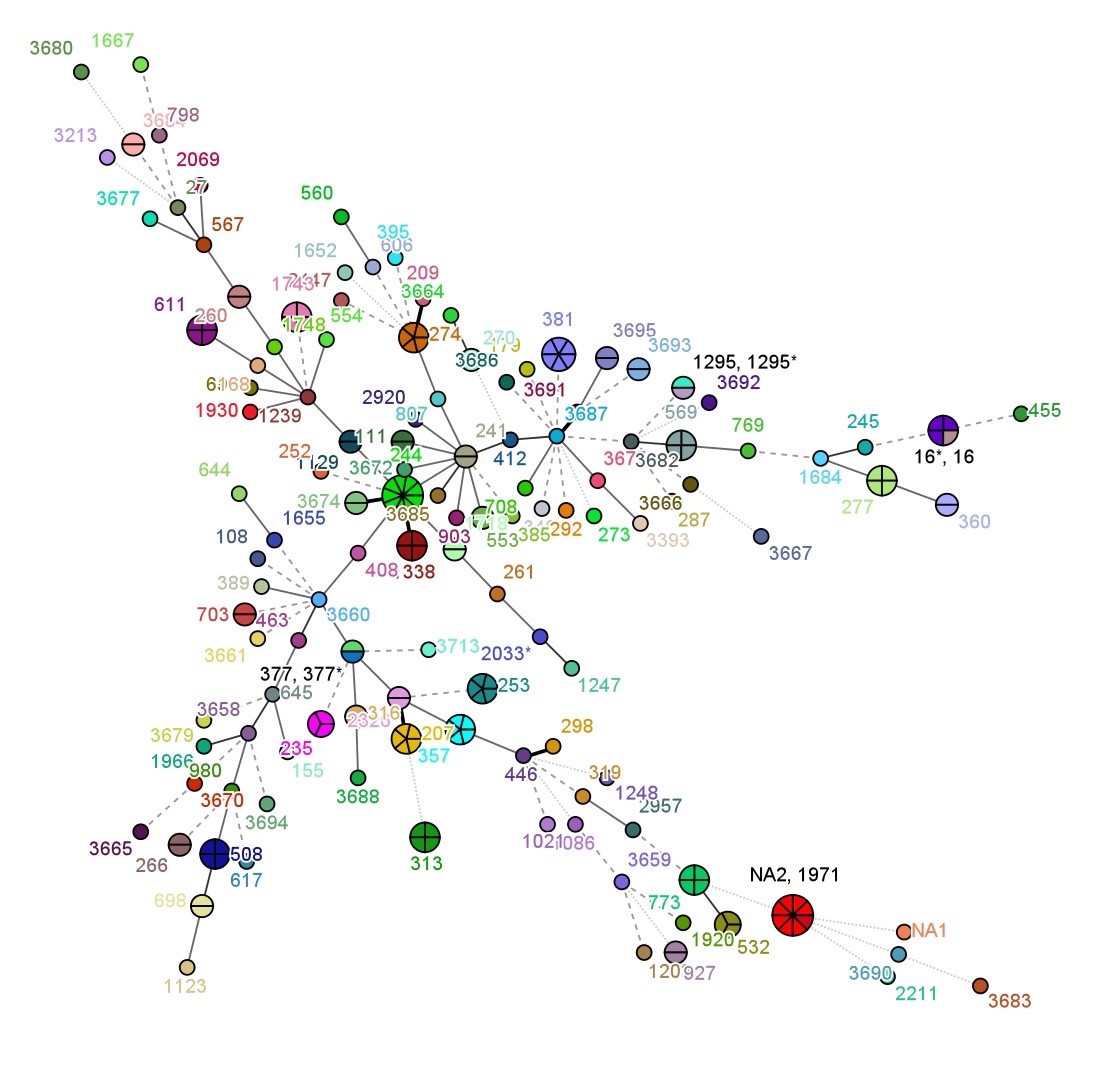


**Supplementary Figure S1. Minimum spanning tree (MST) of MLST data of 212*P. aeruginosa* human isolates collected from 2018 to 2020 in Guangdong Province, China.** Each color represents one sequence type. Circles represent isolates, and the size of the circle is proportional to the number of isolates (NA-no assigned).
